# Dopamine modulation of aggression

**DOI:** 10.1007/s00213-025-06893-w

**Published:** 2025-09-23

**Authors:** Bing Dai, Dayu Lin

**Affiliations:** 1 Neuroscience Institute, New York University Langone Medical Center, New York, NY USA; 2 Department of Brain and Cognitive Sciences, MIT, Cambridge, MA USA; 3 McGovern Institute for Brain Research, MIT, Cambridge, MA USA; 4 Department of Psychiatry, New York University Langone Medical Center, New York, NY USA; 5 Department of Neuroscience and Physiology, New York University Langone Medical Center, New York, NY USA

**Keywords:** Dopamine, Aggressive behavior, Dopamine receptor antagonist, Neuromodulation

## Abstract

**Rationale:**

Aggression is an innate social behavior prevalent across animal species. However, in modern human society, inter-personal aggression is considered disruptive and detrimental to both families and communities. Clinically, antipsychotics, which primarily target dopamine (DA) receptors, have been widely used to suppress hyper-aggression. However, the mechanisms underlying the effect of the antipsychotics remain incompletely understood.

**Objectives:**

We reviewed key steps in brain DA synthesis and summarized genetic and pharmacological evidence supporting the role of the mesolimbic DA system in aggression. Next, we discussed recent circuit studies that elucidate the DA action in modulating aggression-related brain regions. These lines of evidence collectively suggest that DA acts on different brain regions to facilitate aggression and self-learning, and signals the valence of the fighting experience.

**Supplementary Information:**

The online version contains supplementary material available at 10.1007/s00213-025-06893-w.

## Introduction

Aggression is an innate behavior essential for competing for mating opportunities, defending territory, securing resources, and protecting oneself and one’s family (Box 1). It is considered a part of the reproductive behavior repertoire and is prevalent across species, including humans (Tinbergen 1968; Lischinsky and Lin 2020). Given its critical role in individual survival, aggression is driven by a developmentally wired subcortical circuit (Lischinsky and Lin 2020; Wei et al. 2021; Mei et al. 2023). This circuit’s activity is further shaped by multiple neuromodulators, which influence specific neuronal populations within aggression-related pathways (de Almeida et al. 2005; Miczek et al. 2002). Dopamine (DA) has long been implicated in the modulation of aggressive behaviors. Drugs antagonizing DA receptors are the most widely used pharmacological treatment for suppressing hyperaggression in clinical settings (Yudofsky et al. 1987; van Schalkwyk et al. 2018). This review aims to summarize many lines of evidence supporting DA’s modulation of aggression and to discuss our current understanding of the neural circuit mechanisms underlying such modulation. We will first introduce the key proteins involved in DA synthesis and genetic studies, indicating their functions in aggression. Then, we will summarize various pharmacological studies that demonstrate the impact of DA signaling on aggression. Lastly, we will discuss the aggression circuits that are modulated by DA and ultimately influence the behaviors.

**Table:** Box 1 Different types of aggression and behavioral procedures in mice

Aggressive behavior can be divided into reactive and proactive aggression (Golden et al. 2019b). Reactive aggression is a defensive, impulsive response to a perceived threat and represents an innate, unconditioned form of aggression. One widely used behavioral procedure to induce reactive aggression in mice is social isolation (Takahashi and Miczek 2014). Male mice are single-housed for several weeks before a group-housed conspecific male intruder is introduced into the resident’s cage. Under this resident-intruder task, most of the resident mice gradually develop aggression and initiate attacks toward the intruder male mice after repeated intruder exposures. Maternal aggression represents another form of reactive aggression. Female mice are generally non-aggressive under normal conditions; however, they become highly aggressive during the postpartum period, displaying vigorous defensive attacks to protect their offspring (Hashikawa et al. 2018). Reactive aggression also varies from species to species. More details can be found in our previous review (Lischinsky and Lin 2020).
Proactive aggression, by contrast, is a planned and instrumental form of aggression characterized by self-motivated aggression seeking. This type of aggression is more commonly observed in humans but can also be modeled in mice using Pavlovian and operant-based tasks (Golden et al. 2019b; Wrangham 2018). In Pavlovian conditioning procedures, socially isolated aggressive mice are placed in a two-chamber setup: one where they can interact with and attack an intruder male, and another without an intruder. After repeated aggressive encounters, the aggressive mice develop a conditioned place preference for the intruder-paired chamber, indicating the rewarding nature of aggression (Aleyasin et al. 2018; Golden et al. 2016). In operant tasks, aggressive mice are trained to perform an arbitrary action, such as lever pressing, to gain access to an intruder for attack, allowing the isolation of the seeking phase of aggression (Falkner et al. 2016; Golden et al. 2019a). A comprehensive review of the behavioral procedures used to study proactive aggression in mice is available in (Golden et al. 2019b).
Studies have indicated that dopamine (DA) is involved in both reactive and proactive forms of aggression. However, the majority of research to date has focused on reactive aggression, which will be the primary focus of this review. We will briefly discuss the DA function in proactive aggression in the neural circuits section.

## Systematic studies on DA and aggression

### DA metabolic and signaling pathways

**Fig. 1:**
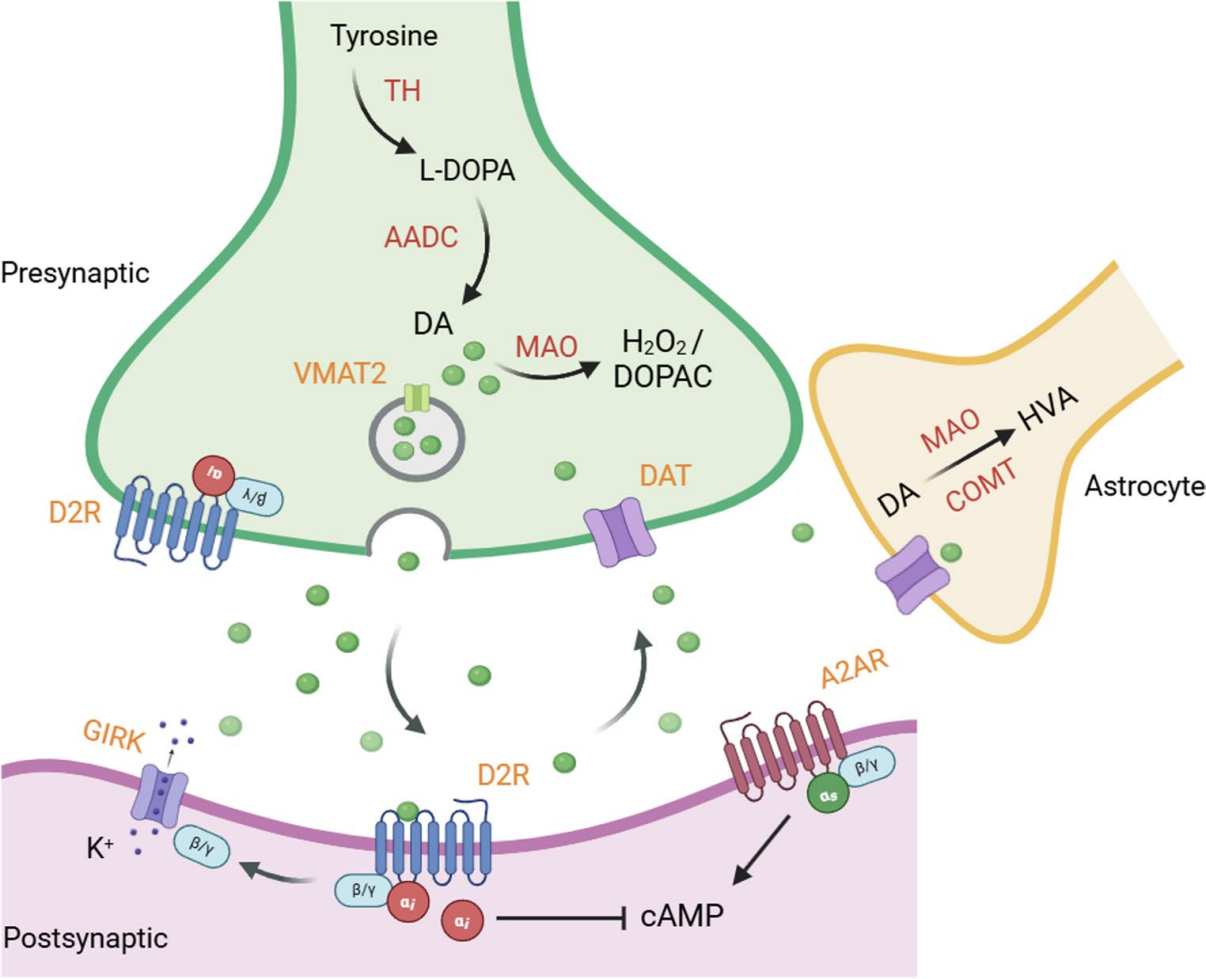
Schematic Illustration of Dopamine Metabolism in the Brain. Tyrosine is converted into L-DOPA by the rate-limiting enzyme TH, and L-DOPA is subsequently decarboxylated to DA by AADC. DA is then packaged into presynaptic vesicles via the VMAT2 and released into the synaptic cleft upon neuronal activation. In this schematic, only D2 receptors are depicted. Once released, DA binds to postsynaptic D2 receptors and/or presynaptic D2 autoreceptors. Activation of these receptors triggers the dissociation of the Gαi subunit, which inhibits adenylyl cyclase activity and reduces cellular excitability, while the Gβγ subunits activate GIRK channels, inducing potassium efflux. Additionally, activation of adenosine A2A receptors antagonizes the effects of D2 receptor signaling. Finally, DA is either reuptaken by the DAT for subsequent release or degradation by metabolic enzymes. TH, tyrosine hydroxylase; L-DOPA, L-3,4-dihydroxyphenylalanine; AADC, L-aromatic amino acid decarboxylase; VMAT2, vesicular monoamine transporter 2; MAO, monoamine oxidase; DOPAC, 3,4-dihydroxyphenylacetic acid; HVA, homovanillic acid; D2R, D2 receptor; DAT, dopamine transporter; GIRK, G protein-gated inwardly rectifying potassium; A2AR, adenosine A2A receptors; cAMP, cyclic adenosine monophosphate. Figure was created using BioRender (https://biorender.com)

DA is primarily synthesized by dopaminergic cells in the midbrain and hypothalamus from the amino acid tyrosine (Björklund and Dunnett 2007). Tyrosine is first converted into L-3,4-dihydroxyphenylalanine (L-DOPA) by the rate-limiting enzyme tyrosine hydroxylase (TH). L-DOPA is then decarboxylated to DA by L-aromatic amino acid decarboxylase (AADC), an enzyme also involved in serotonin (5-HT) synthesis (Himmelreich et al. 2019; Klein et al. 2019). Once synthesized, DA is packaged and stored in presynaptic vesicles by the vesicular monoamine transporter 2 (VMAT2), preparing it for release during neurotransmission (Klein et al. 2019) (Fig. 1). In addition to dopaminergic cells, DA can also be synthesized in norepinephrinergic cells, where it serves as a precursor for norepinephrine (NE) through catalyzation of dopamine β-hydroxylase (DBH) (Himmelreich et al. 2019). Interestingly, DA can also be released from these noradrenaline terminals in regions such as the prefrontal cortex and dorsal hippocampus (Devoto et al. 2001, 2005; Kempadoo et al. 2016).

Once DA is released in the synaptic cleft, it binds to DA receptors and modulates cell excitability through G proteins (Fig. 1). DA receptors can be classified into two subfamilies: D1-like and D2-like receptors. D1-like receptors, including D1 and D5, are coupled with Gαs/olf subunit of G proteins. When DA binds to these receptors, the Gαs/olf subunit dissociates from Gβγ subunits and leads to the activation of adenylyl cyclase, the elevation of cyclic AMP (cAMP) levels, and ultimately, the increase of cell excitability (Beaulieu and Gainetdinov 2011). In contrast, D2-like receptors (D2, D3, and D4) are linked to the Gαi/o subunit, which can inhibit adenylyl cyclase activity and decrease the excitability of the cells (Beaulieu and Gainetdinov 2011). The activation of the D2-like receptor also releases the Gβγ subunits of G proteins. These subunits can quickly traffic and bind to G protein-gated inwardly rectifying potassium (GIRK) channels on the cell membrane, reducing the membrane potential in a few milliseconds through potassium influx (Neve et al. 2004) (Fig. 1).

Among all DA receptors, D2 receptors are the ones most closely linked to aggression in humans and possess some unique features that might be relevant to their roles in aggression (Brizer 1988; Yudofsky et al. 1987). D2 receptors are encoded by the Drd2 gene, which contains five introns that enable the generation of two splice variants: D2-short (D2S) and D2-long (D2L) (Beaulieu and Gainetdinov 2011). The D2L isoform includes an additional 29 amino acids inserted into the third cytoplasmic loop of the protein. D2L is predominantly expressed postsynaptically in the striatum, whereas D2S mainly functions as an autoreceptor located at the presynaptic terminals of dopaminergic neurons (Cases et al. 1995; Usiello et al. 2000). Furthermore, D2 receptors are often co-expressed with adenosine A2A receptors (A2A) in certain cell types, e.g., medium spiny neurons of the striatum (Schiffmann et al. 2007). This co-expression has led many studies to utilize A2A-cre transgenic mice, which express the Cre recombinase under the control of the adenosine A2A receptor promoter, to specifically target D2R-expressing medium spiny neurons (MSNs) in the striatum (Markowitz et al. 2018; Tritsch et al. 2012; Zachry et al. 2024), which eliminates the influence from D2R-expressing cholinergic interneurons (Puighermanal et al. 2020). A2A receptor activation antagonizes the effects of D2 receptors by increasing cAMP levels, thereby fine-tuning intracellular signaling pathways (Azdad et al. 2009; Schiffmann et al. 2007) (Fig. 1).

After DA dissociates from DA receptors, it gets reuptaken by dopamine transporter (DAT) at presynaptic terminals or nearby microglia for recycling (Meiser et al. 2013). Finally, DA is degraded by monoamine oxidase A (MAO-A) and catechol-O-methyltransferase (COMT) into homovanillic acid, which dissolves in cerebrospinal fluid and is eventually cleared from the brain (Himmelreich et al. 2019; Meiser et al. 2013) (Fig. 1).

In summary, many enzymes are involved in DA metabolism, and DA interacts with various receptors and signaling molecules to modulate cellular activities. The following two sections provide a detailed review of genetic and pharmacological studies, examining how alterations in these DA-related pathways influence aggressive behavior in human and animal models.

## Genetic mutations

### DA synthesis and release

Genetic mutations in proteins critical for DA synthesis and release, such as TH and VMAT2, result in severe developmental failures in both humans and mice, making aggression evaluation difficult (Kobayashi et al. 1995; Zhou et al. 1995; Wang et al. 1997; Kim et al. 2000; Caine et al. 2017). For example, disrupting the TH gene completely depletes DA in mice, leading to severe cardiovascular dysfunction and perinatal lethality (Kobayashi et al. 1995; Zhou et al. 1995; Zhou and Palmiter 1995). Although administering L-DOPA to pregnant females can rescue TH-deficient (TH -/-) mice in utero, these mice fail to survive past weaning without continued postnatal L-DOPA supplementation (Zhou et al. 1995; Zhou and Palmiter 1995). Szczypka et al. reported that adult male TH-/- mice exhibited shorter attack latencies and slightly prolonged attack durations during 10-minute resident-intruder tasks (RITs) following two weeks of social isolation (Szczypka et al. 1998) (Table 1). However, TH -/- mice in this study required daily L-DOPA injections to maintain basic physiological functions and normal behaviors. L-DOPA itself was found to promote aggression in TH -/- mice in their study, making the interpretation of their aggressive behavior complicated (Szczypka et al. 1998). In humans, only individuals with less severe mutations in the TH gene can survive (Nagatsu et al. 2019). However, even in these cases, most exhibit a progressive motor dysfunction starting shortly after birth or during early childhood, which is often accompanied by developmental and intellectual disabilities (Hoffmann et al. 2003; Willemsen et al. 2010).

**Table 1:** Aggression and genetic mutations in DA metabolic pathways

Gene Name	Function	Subject	Behavioral Task	Finding about aggression	Notes	Reference
TH	Convert Tyrosine to L-DOPA	129 Sv/CPJ X C57BL/6 TH-/- Male mice	Single-housed for 2 weeks, treated daily with 50 mg/kg L-DOPA, RITs for 10 mins	DA -/- male with daily L-DOPA supply showed reduced attack latency and increased attack duration, compared with wild-type (WT) male mice	Without supplement of the L-DOPA, the animal die after brith	Szczypka et al., 1998
D2L	Long isoform of D2 receptor	129 Sv/CPJ X C57BL/6 D2L-/- Male mice	Single-housed for 4 weeks, RITs for 5 mins	Most D2L-/- male failed to attack intruders over three-day tests		Vukhac et al., 2001
Girk	GIRK subunit 1 to form GIRK channel that can instantaneously suppress cellular acitivty upon D2R activaiton	C57BL/6J Girk1-/- Male mice	Single-housed for 4-5 weeks, RITs for 15 mins	A comparable percentage of Girk1-/- male mice showed aggression but with longer latency and shorter attack duration		Kim et al., 2012
A2A	Adensosine receptor that agantonizes D2R's function	129 Sv/CP X CD1 A2A-/- Male and Female mice	Single-housed for 4 weeks, RITs for 5 mins once per week	Most A2A -/- male mice showed aggression with shorter latency, longer duration, and more freqeunt tail rattles; No difference in females		Ledent et al., 1997
DAT	DA transporter to recycle DA from synaptic cleft	129 Sv/CPJ X C57BL/6 DAT-/- Male mice	Single-housed for 2 weeks, RITs for 10 mins for 6 days	Attack frequency was generally higher than in WT controls in most of the test days.		Rodriguiz et al., 2004
MAO A	Degrade DA	C3H/HeJ MAO A -/- Male mice	Housed with female or single-housed for 4 weeks, RITs for 10 mins	Latency to first attack in both conditions dramatically decreased compared with WT control		Cases et al., 1995
COMT	Degrade DA	COMT +/- and COMT -/- Male mice	Mice from same genotype are matched to perfrom 3-day intermale aggression tests in a neutral cage for 15 min each day	COMT +/- male mice showed the shortest latency to attack and the largest number of attacks during 15 min RITs compared with WT and COMT -/- male.		Gogos et al., 1998

Similarly, mice with complete loss of VMAT2, which is responsible for packaging monoamines (including DA, 5-HT, and NE) into presynaptic vesicles for release, exhibit a dramatic reduction of monoamine concentration in the brain and die within days of birth (Wang et al. 1997). Due to the critical roles of genes involved in DA synthesis and release in development and motor function, investigating their potential links to aggression is challenging in these models.

### D2 Receptors

Among the five subtypes of DA receptors, genetic mutations affecting D2 receptor and its related signaling proteins cause the most consistent changes in aggressive behaviors in both mice and humans (Pavlov et al. 2012; Vukhac et al. 2001) (Table 1).

D2 receptors have two isoforms – D2L and D2S – resulting from alternative mRNA splicing (Beaulieu and Gainetdinov 2011). Genetic disruption of D2L in mice does not significantly affect overall D2 receptor density due to compensatory upregulation of D2S (Wang et al. 2000). Nevertheless, D2L-deficient (D2L -/-) mice exhibit increased sensitivity to quinpirole, a D2-like agonist, and reduced response to haloperidol, a D2-like antagonist (Yudofsky et al. 1987; Eilam and Szechtman 1989; Usiello et al. 2000; Wang et al. 2000). Male D2L -/- mice display reduced social-isolation induced aggression (Vukhac et al. 2001) (Table 1). During RITs conducted over three consecutive days, only a minority of D2L -/- mice (3/17) exhibited aggressive behavior toward conspecific intruders, whereas all wild-type (WT) littermates (12/12) demonstrated aggression (Vukhac et al. 2001).

In humans, various polymorphisms in the Drd2 gene have been identified over the past few decades (Zou et al. 2012). While many studies have reported correlations between specific Drd2 polymorphisms and hyperaggressive phenotypes in different populations, the findings often lacked consistency (Beaver et al. 2007; Guo et al. 2007; Zai et al. 2012; Butovskaya et al. 2013; Della Torre et al. 2018; 18 et al., 2018; Halicka-Masłowska et al. 2021) (Table 2). Among these, the TaqIA single-nucleotide polymorphism (SNP) of the Drd2 gene stands out as a relatively reliable predictor of hyperaggression (Guo et al. 2007; Zai et al. 2012; Butovskaya et al. 2013; Beaver et al. 2007; 18 et al., 2018). This TaqIA polymorphism is located in the 3′ untranslated region of the Drd2 gene. Individuals carrying the A1 allele of this polymorphism have a C-to-T point mutation (TCGA to TTGA), which leads to a significant reduction in D2 receptor availability in the striatum compared with individuals with the A2/A2 genotype (Noble et al. 1991; Thompson et al. 1997; Pohjalainen et al. 1998). Several studies across diverse populations have consistently found that individuals, particularly male adolescents, with the A1/A2 genotype are more prone to show aggressive behaviors, such as anger and violent delinquency, while the correlation in female subjects shows less consistency (Guo et al. 2007; Zai et al. 2012; Butovskaya et al. 2013).

**Table 2:** Human aggression and Drd2 gene polymorphisms

Subject	Defination of aggression	Gene						Reference
		A-241G (rs1799978)	TaqIA (rs1800497)	rs1079598	C957T (rs6277)	TaqID (rs1800498)	−141C Ins/Del (rs1799732)	
144 children from the Greater Toronto Area	Above 90th percentile on the aggression subscales of both the Child Behavior Checklist and the Teacher's Report Form	G allele is associated with aggression. More significant if only include male data	A1/A2 and A1/A1 genotype are overrepresentated in aggressive children. More significant if only include male data	CC genotype is overrepresented in aggressive children. More significant if only include male data	No assocation	No assocation	No assocation	Zai et al., 2012
Sibling subsample of more than 2,500 participants in the National Longitudinal Study of Adolescent Health.	Violent delinquency	NA	The violent delinquency is more frequent in A1/A2 genotype in male. No assocation in female.	NA	NA	NA	NA	Guo et al., 2007
138 adult Datoga men	Self-rated aggression scores were obtained using Buss and Perry’s Aggression Questionnaire (AQ)52	NA	Anger is highly associated with the genetype of A1/A2 and A2/A2.	NA	NA	NA	NA	Butovskaya et al., 2013
872 male participants from the National Longitudinal Study of Adolescent Health	Self-report questionnaires that tapped adolescent conduct disorder and adult antisocial behavior	NA	No assocation, unless included the gene x gene interaction with Drd4	NA	NA	NA	NA	Beaver at al. 2007
820 healthy unrelated Hadza and Datoga individuals	Self-rated scores of aggression were collected using Buss and Perry's Aggression Questionnaire.	NA	No assocation	NA	NA	NA	NA	Sukhodolskaya et al. 2018
Participants were 144 adolescents with conduct disorder recruited at the youth socio-therapy centre.	The Buss-Perry Aggression Questionnaire (BPAQ) was administered to record the level of aggression	NA	NA	NA	No assocation	NA	NA	Halicka-Masłowska et al. 2021.
Children and adolescents between 8 and 20 years old who were clinically followed-up.	Behavioral characteristics of children and adolescents based on an inventory of the Child Behavior Checklist	No assocation	NA	NA	CT and TT genotypes are siginifcantly associated with defiant and oppositional problems.	NA	NA	Della Torre et al. 2018

How does reduced D2 receptor availability in individuals with the TaqIA polymorphism contribute to hyperaggressive phenotypes? Clinically, hyperaggressive patients are often treated with D2 receptor antagonists to suppress aggression (McDougle et al. 1998; Volavka et al. 2004b), seemingly contradicting the notion that reduced D2 receptor availability could drive aggressive behavior. Follow-up studies have partially resolved this discrepancy. Positron emission tomography studies found that heterozygous carriers of the A1 allele have a significantly higher L-DOPA uptake rate in the putamen than non-carriers, despite lower D2 receptor availability (Thompson et al. 1997; Laakso et al. 2005). This effect likely results from reduced D2 autoreceptors, causing increased DA synthesis. Additionally, A1 allele carriers show slightly increased expression of D2 receptors in extrastriatal areas (Hirvonen et al. 2009). These findings suggest increased aggressive behaviors observed in A1 allele carriers may arise from enhanced extrastriatal D2 receptor signaling. Supporting this hypothesis, another Drd2 polymorphism, C957T, which increases D2 receptor expression in the striatum but decreases in extrastriatal regions (Hirvonen et al. 2004, 2009), does not show a consistent correlation with overt aggression in human studies (Zai et al. 2012; Della Torre et al. 2018; Halicka-Masłowska et al. 2021).

D2 receptors are coupled with GIRK channels to facilitate rapid inward potassium currents upon binding DA (Beaulieu and Gainetdinov 2011). Activation of GIRK channels quickly hyperpolarizes the cell membrane, suppressing neuronal activity. This coupling is well known in midbrain DA cells, where it plays a key role in regulating presynaptic DA release via D2 autoreceptors (Ford 2014). Recent studies have identified a similar D2 receptor-GIRK association at postsynaptic terminals in the dorsal lateral septum (dLS) of mice (Mahadevia et al. 2021; Dai et al. 2025). Interestingly, male mice lacking Girk1, a GIRK channel subunit predominantly expressed in the dLS and cortical areas but not in the midbrain or striatum (Lein et al. 2007; Yao et al. 2023), become less aggressive in RITs following social isolation (Kim et al. 2012) (Table 1). The Girk1 knock-out mice show a longer latency to initiate their first attack and a reduced attack frequency in the RITs compared with the WT controls (Kim et al. 2012). Thus, D2 receptor signaling may facilitate aggression by activating GIRK channels.

A2A receptors may influence aggression indirectly through their close interactions with D2 receptors. In the striatum, A2A receptors are colocalized with D2 receptors in MSNs and can antagonize D2 receptor effects by activating Gαq subunits and increasing cAMP production (Beaulieu and Gainetdinov 2011). In the LS, A2A receptors are sparsely expressed in roughly 1% of cells (Wang et al. 2022, 2023), but can exert strong inhibitory effects on surrounding cells, many of which express D2 receptors (Mahadevia et al. 2021; Yao et al. 2023; Dai et al. 2025). Local infusion of CGS21680, an A2A receptor agonist, in the LS enhances this inhibition and significantly reduces c-Fos expression (Wang et al. 2023). Thus, A2A receptors can antagonize the effect of D2 receptors both intracellularly and intercellularly, indirectly regulating aggression. Indeed, knocking out A2A receptors significantly increases the aggression in male mice during RITs, while leaving females unaffected (Ledent et al. 1997) (Table 1). More specifically, four weeks after single housing, 80% of A2A -/- males initiated their first attack within 3 min of their first social encounter, whereas less than 20% of WT males displayed aggression during the entire 5-minute test period.

### DA degradation and recycling

Genes involved in DA degradation and recycling are strongly associated with hyperaggressive phenotypes in both humans and mice (Brunner et al. 1993a, b; Cases et al. 1995; Gogos et al. 1998; Rodriguiz et al. 2004; Volavka et al. 2004a). DA is primarily degraded by MAOA within the mitochondria and COMT in the astrocytes (Meiser et al. 2013) (Fig. 1). Deficiency in either gene has been closely linked to overt aggressive conduct, particularly in males. For example, MAOA was the first gene identified that can directly cause impulsive aggression in males (Brunner et al. 1993a, b). Brunner and colleagues discovered a point mutation in the 8th exon of the MAOA gene located on the X chromosome in a large Dutch kindred. This SNP caused early termination of the mRNA translation, resulting in a severe reduction of MAOA enzymatic activity (Brunner et al. 1993b). All male carriers in this family exhibited markedly increased frequency of aggressive and violent behaviors. In mice, genetically knocking out MAOA successfully replicates the hyperaggressive phenotype observed in humans (Cases et al. 1995) (Table 1). These knock-out mice exhibit significantly more frequent aggression between cagemates than controls and a markedly reduced latency to initiate attacks during their first RITs. Additionally, the dopamine metabolite, 3,4-dihydroxyphenylacetic acid (DOPAC), is decreased drastically in three-month-old knock-out mice, accompanied by an increase in extracellular DA concentration (Cases et al. 1995).

The dopamine transporter (DAT), the primary membrane protein responsible for DA reuptake, also profoundly affects aggression. In both mice and rats, genetic knock-out of DAT dramatically increases basal extracellular DA concentration by more than 5-fold in DAT -/- animals compared with WT controls (Giros et al. 1996; Jones et al. 1998; Leo et al. 2018) (Table 1). Interestingly, while striatal DA release upon electrical stimulation is reduced substantially in DAT -/- rodents, once released, DA remains in the extracellular space about 100 times longer than in WT mice. This prolonged DA presence likely amplifies DA signaling, contributing to behavioral changes. Male DAT -/- mice exhibit increased aggressiveness during RITs after social isolation compared with WT controls (Rodriguiz et al. 2004). Additionally, these mice showed an unstable social rank across consecutive days in tube tests, suggesting similar social dominance among DAT mutant animals (Rodriguiz et al. 2004).

## Pharmacology

### Drugs disrupting DA synthesis and release

Drugs that inhibit DA synthesis can effectively reduce aggression in male mice. α-Methyl-p-tyrosine (AMPT) is a competitive inhibitor of TH, which can block the conversion of tyrosine to L-DOPA (Table 3). Although it takes hours to show an effect, it can effectively reduce the DA storage in DA cells (Watanabe et al. 2005). Serova et al. reported that 7 h after intraperitoneal (i.p.) injection of AMPT, DA concentrations in the brain hemispheres and brainstem were significantly reduced in mice (Serova and Naumenko 1996). Behaviorally, 8 out of 9 dominant male mice, identified by their victories in RITs prior to treatment, displayed significantly fewer attacks and lost their dominant status after AMPT administration (Serova and Naumenko 1996). AMPT treatment also caused a dramatic increase in the latency to initiate their first attack compared with vehicle-treated controls. This reduction in aggression was not due to compromised general locomotion, as AMPT-treated mice exhibited similar motor activity to controls in open-field tests (Serova and Naumenko 1996).

**Table 3:** Aggression and chemicals to reduce DA

Chemical	Function	Subject	Behavioral Task	Results	Reference
α-Methyl-p-tyrosine	TH inhibitor	Male mice from different strains	Single-housed for 5 days, RITs for 3 hours with four 20-min observation periods	Aggression decrease.	Serova and Naumenko, 1996
CGS 10746B	DA release inhibitor	Male OF1 mice	Single-housed for 4 weeks, RITs for 10-min in a neutral cage	Aggression decrease. Mice with long attack latency showed a stronger response than those that had a short attack latency	Felip et al., 2001
6-OHDA	Kill DA cells	Cats of both sexes	Home cage aggression and aggressive behavior towards human	Aggression increase after first several doses of 6-OHDA infusion, then aggression decrease	Beleslin et al., 1981, 1986
6-OHDA	Kill DA cells	Male CF1 mice with high isolation-induced fighting tendencies	Single-housed or pair-housed with a female after weaning, RITs for 10 mins	Fighting tendency decrease.	Crawley and Contrera, 1976
6-OHDA	Kill DA cells	Male albino mice	Single-housed for 3-5 weeks, RITs for 4 mins with three repeats in a week	Increase aggression in non-aggressive mice, but no change in experienced aggressors	Pöschlová et al., 1976
6-OHDA	Kill DA cells	Male Long Evans rats	Social hierachy test in big cohorts (27 male for each treatment)	6-OHDA treated rats started with high hierarchy then gradually decreased to the lowest.	Ellison, 1976
6-OHDA	Kill DA cells	Female Sprague-Dawley rats	Shock-induced aggression and maternal aggression.	Increase both shock-induced and maternal aggression.	Sorenson and Gordon, 1975
6-OHDA	Kill DA cells	Male Wistar rats	Tail-clamp induced aggression	Increase tail-clamp induced aggression.	Mine et al., 1981
6-OHDA	Kill DA cells	Male Sprague-Dawley rats	Shock-induced aggression	Increase shock-induced aggression.	Eichelman et al., 1972
6-OHDA and DA	Kill DA cells	Male Sprague-Dawley rats	Shock-induced aggression	Both 6-OHDA and DA increase shock-induced aggression	Geyer and Segal, 1974
6-OHDA	Kill DA cells	Male Sprague-Dawley rats	Shock-induced aggression	Increase shock-induced aggression.	Thoa et al., 1972
6-OHDA	Kill DA cells	Male Wistar rats	Shock-induced aggression	6-OHDA infusion inventral tegmental area (VTA) decrease shock-induced aggression.	Puciłowski et al. 1982
6-OHDA	Kill DA cells	Male Swiss mice	6-OHDA neontal lesion at postnatal day 5, RITs at postnatal day 42	6-OHDA infusion at P5 increase aggression during adolescence (P42)	Bouchatta et al. 2018

Pharmacological inhibition of DA release also influences intermale aggression. CGS 10746B, a DA release inhibitor, can suppress DA cell firing rates without altering DA metabolism or occupying D2 receptors (French and Witkin 1993) (Table 3). i.p. injection of the CGS 10746B, at a dose that did not affect general locomotion, effectively reduced intermale aggression after social isolation (Felip et al. 2001). Interestingly, the anti-aggression effect of CGS 10746B was much stronger in the mice had longer attack latency (> 3 min) compared with those had shorter latency (< 2 min) (Felip et al. 2001). Typically, male mice that are more aggressive or have more experience with aggression exhibit shorter latencies to attack (Yan et al. 2024). These results suggest that the aggressiveness level of mice may influence the effectiveness of the drug in modulating aggressive behavior.

### 6-Hydroxydopamine

6-Hydroxydopamine (6-OHDA) is a neurotoxin widely used to model Parkinson-like symptoms in rodents due to its ability to selectively induce oxidative and metabolic stress in DA cells (Simola et al. 2007). Because 6-OHDA cannot readily cross the blood-brain barrier, its administration requires direct intraventricular infusion for widespread effects or targeted injection into specific brain regions. Once inside the brain, 6-OHDA enters DA cells through DAT, and it can rapidly trigger the degradation of DA axons at the injection site within a few hours (Stott and Barker 2014). However, the 6-OHDA-induced apoptosis of the midbrain DA cells is a slower process, taking 1 ~ 2 weeks to reach a steady state (Stott and Barker 2014). The progressive loss of DA innervation leads to complex effects on aggressive behavior in animals (Table 3).

Intraventricular infusion of 6-OHDA in adult animals produces biphasic effects on aggressive behaviors (Crawley and Contrera 1976; Ellison 1976; Pöschlová et al. 1976; Beleslin et al. 1981, 1986). For example, Beleslin and colleagues performed daily intraventricular infusions of 6-OHDA in group-housed cats and observed a transient increase in spontaneous aggression towards cagemates after the first 2–3 infusions (Beleslin et al. 1981, 1986). However, with repeated administration, aggressive behavior typically decreased or disappeared entirely (Beleslin et al. 1981, 1986). This reduction in aggression was accompanied by a gradual decline in general locomotor function. Similar biphasic changes in aggression were observed in rats. Elison infused saline or 6-OHDA intraventricularly in rats for three consecutive days and monitored their behaviors for the following 50 days (Ellison 1976). In the first few days after the treatment, the number of violent fighters in the 6-OHDA-treated groups increased more compared with saline controls. However, within a month, the hierarchy positions of the 6-OHDA-treated rats, determined by victories during aggressive encounters, declined dramatically and never recovered (Ellison 1976). In mice, the effects of 6-OHDA on aggression exhibit some differences compared with other species (Beleslin et al. 1981; Ellison 1976; Pöschlová et al. 1976). 6-OHDA infusion induced aggressive behavior in previously non-aggressive, single-housed male mice (Pöschlová et al. 1976). However, in mice that were already aggressive, 6-OHDA treatment did not significantly alter their overall aggressive behavior (Pöschlová et al. 1976).

In contrast, painful stimuli such as foot shock and tail pinch reliably elicit a higher frequency of attacks in 6-OHDA-treated mice and rats, even several weeks after the last 6-OHDA infusion (Eichelman et al. 1972; Thoa et al. 1972; Geyer and Segal 1974; Sorenson and Gordon 1975; Mine et al. 1981). In the foot shock-induced aggression, rodents typically receive 1–2 doses of 6-OHDA infusion several weeks before the aggression test. During the test, 6-OHDA-treated rodents, along with either their cagemates or group-housed male intruders, are introduced into a foot shock chamber where both animals receive simultaneous shocks. The pain stimulus triggers fighting between the two animals, and the 6-OHDA-infused rodents consistently displays more attacks compared with the saline controls (Eichelman et al. 1972; Thoa et al. 1972; Geyer and Segal 1974).

Why does a neurotoxin that kills DA cells and depletes DA in the brain, to some extent, facilitate aggression? These observations contradict genetic and pharmacological evidence supporting DA’s role in promoting aggression (Brizer 1988; Freudenberg et al. 2016; Vukhac et al. 2001). One possible explanation is that DA depletion caused by intraventricular infusion of 6-OHDA leads to hyperactivation of aggression-related neural circuits. Several studies have demonstrated that after 6-OHDA lesions, striatal D1R-expressing cells show a stronger response to DA, and D2R-expressing cells become more reactive to glutamate transmission (Feyder et al. 2011; Ryan et al. 2018; Spigolon and Fisone 2018; Mariani et al. 2019). Additionally, VTA DA cells, which are more relevant to aggressive behaviors (Yu et al. 2014; Mahadevia et al. 2021; Dai et al. 2025), show greater resilience to 6-OHDA compared with substantia nigra SNc DA cells (Pacelli et al. 2015; Balzano et al. 2024). A single dose of 6-OHDA is often insufficient to fully eliminate VTA DA cells (Bouchatta et al. 2018). Moreover, direct infusion of 6-OHDA at the VTA decreases rather than increases shock-induced aggression (Puciłowski et al. 1982). Taken together, 6-OHDA infusion may induce hyperactivation of downstream DA receptor-expressing cells, while the DA supply related to aggression remains relatively intact. This may explain why 6-OHDA induces biphasic effects on aggressive behaviors (Crawley and Contrera 1976; Ellison 1976; Pöschlová et al. 1976; Beleslin et al. 1981, 1986). During the early stages of 6-OHDA treatment, DA depletion may enhance aggression due to heightened reactivity in downstream circuits. However, as the animal receives multiple doses of 6-OHDA and mesolimbic dopaminergic cells undergo complete degeneration, aggressive behaviors diminish and eventually disappear.

It is important to acknowledge that although the social isolation-induced aggression and pain-induced aggression are both classified as forms of reactive aggression, they may involve distinct neural mechanisms. Social isolation-induced aggression has been studied extensively over the past several decades, resulting in a relatively well-understood set of neural circuits (Lischinsky and Lin 2020; Mei et al. 2023). In contrast, the mechanisms underlying pain-induced aggression remain poorly understood. How painful stimuli elicits aggressive behavior and how DA is involved in this process require further investigation.

### Stimulants

Stimulants, especially amphetamines, increase extracellular DA levels by inhibiting DA reuptake via the DAT. Studies consistently show that amphetamines alter aggression in a dose-dependent manner across different species, where amphetamines promote aggression at low doses but suppress aggressive behavior at higher doses (Miczek and Tidey 1989) (Table 4). In male stumptail macaques, low doses of amphetamines increased self-directed aggressive behavior, while higher doses decreased it (Peffer-Smith et al. 1983). In socially isolated mice and rats, low doses of amphetamines increased intermale aggression, whereas higher doses caused hyperactivity and decreased aggression (Miczek 1974, 1979; Miczek and Tidey 1989). Interestingly, this dose-dependent effect is also observed in healthy human subjects without prior drug history (Cherek et al. 1986). Notably, this phenomenon is distinct from the drug’s effects on general locomotion. At doses where aggression begins to decrease, voluntary movement continues to either increase or remain at elevated levels (Cherek et al. 1986). This suggests that the DA pathways regulating aggression differ from those controlling general movement. Furthermore, this dose-dependent effect of stimulants appears task-independent, as similar trends have been observed across social isolation-induced and foot shock-induced aggression (Crowley 1972; Miczek and Tidey 1989).

**Table 4:** Aggression and stimulants

Chemical	Function	Subject	Behavioral Task	Results	Reference
Amphetamines	DAT blocker	Male Sprague Dawley rats	Single-house, RITs for 15 mins	Increase aggression at a low dose, decrease at a higher dose	Miczek, 1974
Amphetamines	DAT blocker	Male Long Evans rats	Group-housed, RITs for 5 mins after the first attack	Increase aggression at a low dose, decrease at a higher dose	Miczek, 1979
Amphetamines	DAT blocker	Male stumptail macaque	Self-aggression	Increase self-directed aggression at a low dose, decrease at a higher dose	Peffer-Smith et al. 1983
Amphetamines	DAT blocker	Human males	Aggression response test	Increase aggression response at a low dose, decrease at a higher dose	Cherek et al. 1986
Methamphetamine	DAT blocker	Male Sprague Dawley rats	Shock-induced aggression	Increase aggression at a low dose, decrease at a higher dose	Crowley, 1972; Miczek and Tidey, 1989
Amphetamines	DAT blocker	Male CFW mice	Aggression habituation task: 5-min RITs followed by a 5-min rest period for ten repetitions.	High dose decreases aggression in the first encounter, but maintains the aggressiveness for the later ones	Winslow and Miczek, 1983
Amphetamines	DAT blocker	Male Swiss Webster mice	Pair-housed with female, subjects underwent zero, one, or ten aggressive experience and then ran RITs for 5 mins	Experienced aggressor is more sensitive to the suppressive effect of amphetamines.	Haney et al., 1990

Dr. Klaus Miczek and his colleagues provided further insights into the complexity of amphetamine’s effects on aggression, extending their studies beyond simple dose-dependency using mouse models (Winslow and Miczek 1983; Haney et al. 1990). First, they evaluated how amphetamines influence the habituation of aggression during repeated social exposure to the same intruders (Winslow and Miczek 1983). They found that, while high doses of amphetamine decreased the attack duration during the initial social encounter, they also disrupted the habituation-induced aggression level decrease in later sessions. In this procedure, aggressive male mice were exposed to the same intruder in ten consecutive 5-minute RITs with 5-minute rest intervals. In saline-treated controls, attack frequency and sideway threats exponentially decreased over repeated encounters. However, in amphetamine-treated mice (1–5 mg/kg), although the initial attack frequency was lower than saline controls, the amphetamine-treated mice maintained social interest in the same intruder across subsequent trials, leading to significantly higher cumulative attack frequencies (Winslow and Miczek 1983).

Second, Dr. Miczek and his team found that prior aggression experience alters sensitivity to amphetamines in resident mice (Haney et al. 1990). Low doses of amphetamines increased aggressiveness in male mice with no prior fighting experience. However, after ten prior fighting experiences, the same male mice became hypersensitive to the aggression-suppressing effects of amphetamines. For instance, even a low dose of 0.1 mg/kg, which typically increases aggression in naïve male mice, significantly suppressed aggressive behavior in experienced aggressors (Haney et al. 1990). Importantly, this shift in sensitivity to amphetamines did not affect motor activity, which remained consistent regardless of aggression experience. These findings are one of several lines of evidence that show that aggression experience contributes to how DA modulates male aggression.

### DA receptor agonists and antagonists

DA receptor antagonists, especially D2 receptor antagonists, are among the most frequently prescribed drugs to suppress human aggression (Brizer 1988) (Table 5). For example, both the typical antipsychotic haloperidol and the atypical antipsychotic risperidone have been used for decades to manage aggression in patients with psychosis and other conditions such as bipolar disorder and autism (Thoa et al. 1972; Carlyle et al. 1993; McDougle et al. 1998; Cousins et al. 2009; Calver et al. 2015). Despite their widespread use, debates persist about the mechanisms underlying their efficacy. Recent systematic reviews suggest that the aggression-suppressing effects of these drugs may be secondary to their sedative effects on general locomotion (Khushu and Powney 2016; van Schalkwyk et al. 2018).

**Table 5:** Aggression and DA receport agonists and antagonists

Chemical	Function	Subject	Behavioral Task	Results	Reference
Sulpride	D2 Receptor antagonist	Male mice from various strains	Shock-induced and isolation-induced aggression	Decrease both shock-induced and isolation-induced aggression; block winning-induced aggression elevation	Redolat et al., 1991; Nikulina and Kapralova, 1992; Couppis and Kennedy, 2008
(−)-OSU6162	D2 Receptor antagonist	Male CD-1 mice and female Wistar rats	Single-housed for 7 days, RITs for 15 mins	Decrease both isolation-induced aggression in male mice and estrous cycle-dependent aggression in female rats	Studer et al., 2016
Tiapride	D2 Receptor antagonist	Male OF1 mice	Single-housed for 30 days, RITs for 10 mins in a neutral cage	Decrease aggression at a dose that does not affect locomotion.	Navarro and Manzaneque, 1997
Haloperidol	D2 Receptor antagonist	Male C57BL/6J or Swiss Webster mice and human	Mice: RITs after singly-housed; Human: behavioral evaluation	Decrease aggression in subjects that are new to aggression, but lost its efficacy in well-experienced ones	Kudryavtseva et al.,^1999^; Vukhac et al., 2001; Khushu and Powney, 2016; Volavka et al., 2004b; Fragoso et al., 2016
(-)eticlopride	D2 Receptor antagonist	Male albino Swiss mice	Single-housed for 30 days, RITs for 5 mins	Decrease aggression at a dose that does not affect locomotion.	Ferrari and Giuliani, 1995
Risperidone	D2 Receptor antagonist	Male OF1 or Swiss Webster mice and human	Mice: RITs after singly-housed; Human: behavioral evaluation	Decrease aggressive conducts in both rodents and humans	Rodríguez-Arias et al., 1998; Fragoso et al., 2016; McDougle et al., 1998; Volavka et al., 2004b; McCracken et al. 2002
Apomorphine, bromocriptine	D1/D2 Receptor agonist	Male mice from various strains	Shock-induced aggression	Increase aggression	Nikulina and Kapralova, 1992
PNU91356A	D2 receptor agonist	Male A/J and C57BL/6J mice	Single-housed after 22 days of age, RITs for 5 mins in a neutral chamber	Decrease aggression, increase non-locomotor forms of defensive behavior (e.g. freezing).	Gendreau et al., 2000
Quinpirole	D2 Receptor agonist	Male Swiss Webster and DBA/2 mice	RITs after pair-housed with female or singly-housed	Decrease both locomotor and aggression	Gao and Cutler, 1993; Tidey and Miczek, 1992.
U-99194A	D3 Receptor antagonist	Male OF1 mice	Single-housed for 30 days, RITs for 10 mins in a neutral cage	Increase social investigation, decrease aggression at high dose that also suppresses spontaneous motor activity	Rodríguez-Arias et al., 1999
7-OH-DPAT and PD 128907	D3 Receptor agonist	Male A/J and C57BL/6J mice	Single-housed after 22days of age, RITs for 5 mins in a neutral cage	Decrease aggression, increase non-locomotor forms of defensive behavior and increase the locomotor forms of defensive behavior (e.g. escape, jump) at high doses.	Gendreau et al., 2000
SKF 38393	D1 Receptor agonist	Male mice from various strains	Shock-induced and isolation-induced aggression	Little or no effect to aggression at a dose that does not affect locomotion	Nikulina and Kapralova, 1992; Tidey and Miczek, 1992.
SCH23309	D1 Receptor antagonist	Male mice from various strains	Shock-induced and isolation-induced aggression	Little or no effect to aggression at a dose that does not affect locomotion; block winning-induced aggression elevation	Nikulina and Kapralova, 1992; Couppis and Kennedy, 2008; Bondar’ and Kudryavtseva, 2005
L-741741	D4 receptor antagonist	Male OF1 mice	Single-housed for 30 days, RITs for 10 mins in a neutral cage	Little or no effect to aggression	Navarro et al., 2003

Decades of animal studies on D2 receptor antagonists and their effects on aggression provide valuable insights into the debate (Table 5). Both older and newly developed D2 receptor antagonists, such as haloperidol, risperidone, (−)-OSU6162, and Tiapride, have been shown to effectively suppress social-isolation induced aggression in male mice and rats at doses that do not impair locomotion (Redolat et al. 1991; Ferrari and Giuliani 1995; Navarro and Manzaneque 1997; Kudryavtseva et al. 1999; Fragoso et al. 2016; Studer et al. 2016). These findings strongly support the role of D2 receptor antagonists in managing aggression. However, some studies suggest that the effectiveness of these drugs in suppressing aggression depends on the mice’s prior aggression experience (McMillen et al. 1989; Kudryavtseva et al. 1999). For example, McMillen et al. showed that D2 receptor antagonists fail to suppress aggression unless administered at doses that impair locomotion when mice are extensively trained for aggression—eight sessions over two weeks—before testing (McMillen et al. 1989). Another study, conducted by Kudryavtseva et al., carefully examined the impact of aggression experience on the efficacy of D2 receptor antagonist haloperidol (Kudryavtseva et al. 1999). In novice aggressors, mice new to aggression, haloperidol dose-dependently increased the latency to first attack and decreased total attacking duration 30 min and 24 h after injection. However, neither dose significantly affected any measure of aggressive behavior in the experienced winners, who had been victorious in 20 daily aggressive confrontations (Kudryavtseva et al. 1999).

Similar phenomena have been observed in humans. Antipsychotics, including D2 receptor antagonists, are widely used to manage aggression in psychiatric conditions, such as conduct disorders and autism spectrum disorders (ASD), in pediatric populations (Anderson et al. 1984; McCracken et al., 2002). In a large-scale study of ASD children with severe behavioral problems, nearly 70% of patients responded positively to risperidone. Of these responders, two-thirds maintained the positive effects six months after treatment (McCracken et al., 2002). Note that children with aggressive behavior issues may be considered novice aggressors. In contrast, the effectiveness of antipsychotics in addressing chronic, long-term aggression in adults appears limited and may primarily reflect a general suppression of arousal systems (Khushu and Powney 2016; van Schalkwyk et al. 2018). These clinical findings highlight the critical role of prior aggression experience in determining the effectiveness of antipsychotic treatments. They also suggest that the early stages of aggression development may represent the most effective window for intervention.

In addition to D2 receptor antagonists, the D2 receptor agonists have also been implicated in aggression, although the findings are controversial. Some D2 receptor agonists, such as apomorphine and bromocriptine, increase aggression in both foot-shock and isolation-induced aggressions (McKenzie 1971; Benus et al. 1991; Nikulina and Kapralova 1992). However, other D2 receptor agonists, like quinpirole, have been shown in multiple studies to robustly suppress aggression in male mice (Baggio and Ferrari 1980; Tidey and Miczek 1992; Gao and Cutler 1993; Gendreau et al. 1998). One hypothesis suggests that different D2 receptor agonists may exhibit selectivity for specific subtypes of D2 receptors. For example, quinpirole preferentially activates presynaptic D2S autoreceptors, suppressing presynaptic DA release and downstream D2 receptor signaling (Wang et al. 2000; Usiello et al. 2000). But whether that is the case or not requires further investigation.

Beyond D2 receptors, other D2-like receptors appear to play a minimal role in aggression modulation. A highly specific D3 receptor antagonist, U-99,194 A, has been shown to decrease intermale aggression following systemic administration, but only at high doses that also suppress spontaneous motor activity (Rodríguez-Arias et al. 1999). L-741,741, a selective D4 receptor antagonist, did not demonstrate any anti-aggressive effects (Navarro et al. 2003), suggesting that D3 receptor, but not D4 receptor, is involved in aggression modulation.

The evidence regarding D1 receptors is relatively limited. Some studies have found that D1 receptor agonists and antagonists are ineffective in suppressing aggression (Nikulina and Kapralova 1992; Tidey and Miczek 1992; Rodríguez-Arias et al. 1998; Bondar’ and Kudryavtseva 2005). However, other studies suggest D1 receptor antagonist may play a role in proactive aggression. Systemic injection of SCH 23,390 blocked both aggression self-administration and winning-induced aggression increase (Couppis and Kennedy 2008; Becker and Marler 2015). These findings imply that D1 and D2 receptors may have distinct functions in different types of aggression.

## Neural circuit studies of DA in aggression

Recent technological advancements have significantly improved our ability to investigate DA’s effects on aggression at both cellular and circuit levels. Tools such as optogenetics and chemogenetics now enable researchers to precisely manipulate dopaminergic cells and their downstream targets, allowing for detailed dissection of their roles in aggressive behaviors (Campbell and Marchant 2018; Emiliani et al. 2022).

Moreover, the development of state-of-the-art DA sensors has revolutionized our understanding of DA dynamics (Sun et al. 2018, 2020; Patriarchi et al. 2018, 2020; Zhuo et al. 2024). These advanced tools allow researchers to monitor DA release in real-time, shedding light on how DA signaling downstream of DA cells influences aggression. These technologies provide invaluable insights into the cellular and circuit mechanisms underlying DA’s role in aggression, paving the way for more targeted and effective therapeutic interventions.

### Ventral tegmental area

DA cells in VTA are a major source of DA in the brain and are famous for their functions in reward learning, reinforcement, and motivation (Fields et al. 2007; Bromberg-Martin et al. 2010; Pignatelli and Bonci 2015). Pioneering work by Dr. Mark Ansorge’s lab revealed the role of VTA DA cells in modulating aggression (Yu et al. 2014; Mahadevia et al. 2021). In their study published in 2014, they discovered that blocking the DAT during the peri-adolescence period (P22–41) significantly increased aggressive behaviors in adult male mice due to hypersensitivity of their DA system (Yu et al. 2014). They hypothesized that this alteration in aggression might involve VTA DA cells. To test this hypothesis, they used double transgenic mice (DAT^IRESCre^: ai32) that conditionally express channelrhodopsin-2 (ChR2) in all DAT-expressing cells, which captures nearly all the DA cells in the brain. Then, they implanted optical fibers to specifically target and stimulate VTA DA cells. Optogenetic activation of VTA DA cells in untreated adult mice successfully promoted social isolation-induced aggressive behaviors across all measures, including mounting, tail rattling, and biting (Yu et al. 2014). This is the first direct evidence supporting VTA DA cells’ role in modulating aggression in male mice. In a subsequent publication, they further demonstrated that neighboring DA cells in the substantia nigra pars compacta (SNc) do not contribute to aggressive behavior (Mahadevia et al. 2021). Optogenetic activation of SNc DA cells failed to mimic the effects observed with VTA DA cell activation, highlighting the specific role of VTA DA cells in aggression (Yu et al. 2014; Mahadevia et al. 2021).

In our recent study, we employed chemogenetic techniques to bidirectionally manipulate VTA DA cells and investigate their role in aggression using DREADDs (Roth 2016) and DAT^IRESCre^ transgenic mice (Dai et al. 2025). We showed that activating VTA DA cells significantly increased intermale aggression, while inhibiting these cells markedly suppressed aggressive behaviors. These results are consistent with previous findings and provide additional evidence for the necessity and sufficiency of VTA DA cells in modulating aggression in male mice (Yu et al. 2014; Mahadevia et al. 2021). In addition, we found that activation of VTA DA neurons has a limited or negative impact on maternal aggression in females (Dai et al. 2025). Inspired by previous studies (McMillen et al. 1989; Haney et al. 1990, 1990; Kudryavtseva et al. 1999; Felip et al. 2001), we asked whether aggression experience would influence how DA modulates aggression. We found that the effects of chemogenetic manipulation were only robust in novice aggressors (with less than three days of aggression experience) but not in expert aggressors (with more than eight days of aggression experience) (Dai et al. 2025). We then assessed the necessity of DA in the emergence of aggression by conditionally mutating TH in VTA DA cells. Naive male mice with VTA TH mutagenesis failed to show consistent attack across 8 days of repeated RITs. In contrast, control mice displayed escalated aggression and maintained a consistently high level in subsequent testing days. Interestingly, in expert aggressors, the loss of TH in VTA DA cells did not negatively impact aggression; both TH-KO and control mice continue to show high levels of aggression (Dai et al. 2025). These findings highlight the critical role of DA in the emergence of aggression and emphasize how fighting experience shapes DA’s role in modulating aggression.

VTA DA cells are a highly heterogeneous population where molecularly differentiable subpopulations form distinct projections to downstream areas (Beier et al. 2015; Poulin et al. 2018). In the following sections, we will summarize the current knowledge of each brain region innervated by VTA DA cells, detailing their roles in aggressive behavior and discussing their potential mechanisms.

### Lateral septum

LS is one of the downstream targets of VTA DA cells and receives moderate DA inputs (Beier et al. 2015; Poulin et al. 2018). It plays an important yet complex role in regulating aggressive behavior. Previous studies have demonstrated that the dorsal (dLS) and ventral (vLS) subdivisions of LS work in coordination to modulate intermale aggression (Wong et al. 2016; Leroy et al. 2018; Guo et al. 2023). The dLS receives aggression-related social information from the CA2 subregion of the hippocampus and sends direct inhibition to the vLS, which in turn disinhibits neurons in the ventrolateral part of ventromedial hypothalamus (VMHvl), a region known to promote aggressive behaviors (Lin et al. 2011; Wong et al. 2016; Leroy et al. 2018). Fiber photometry recording showed that both pyramidal cells in CA2 and their terminals projecting to the dLS are activated during attack (Leroy et al. 2018). Furthermore, our recent findings revealed that attacking male intruders robustly increase GCaMP6 signals in dLS cells, supporting its active role in facilitating intermale aggression (Dai et al. 2025).

DA release into the dLS is critical for intermale aggression. Studies by Dr. Mark Ansorge’s lab and our lab demonstrated that optogenetic activation of VTA DA terminals in the dLS promotes aggressive behaviors (Mahadevia et al. 2021; Dai et al. 2025). In contrast, optogenetic inhibition of VTA DA terminals in the dLS, or ablating these terminals using 6-OHDA, significantly diminishes aggressive behaviors in rookie aggressors (Mahadevia et al. 2021; Dai et al. 2025). Interestingly, after animals gain enough aggression experience, we found that the DA modulation in dLS becomes less effective. Using the third-generation fluorescent DA sensor, GRAB_DA3h_, we showed that DA release in the dLS is highly responsive when intruders are present to rookie aggressors. However, as aggression experience accumulates, DA release in the dLS becomes progressively limited (Dai et al. 2025).

**Fig. 2:**
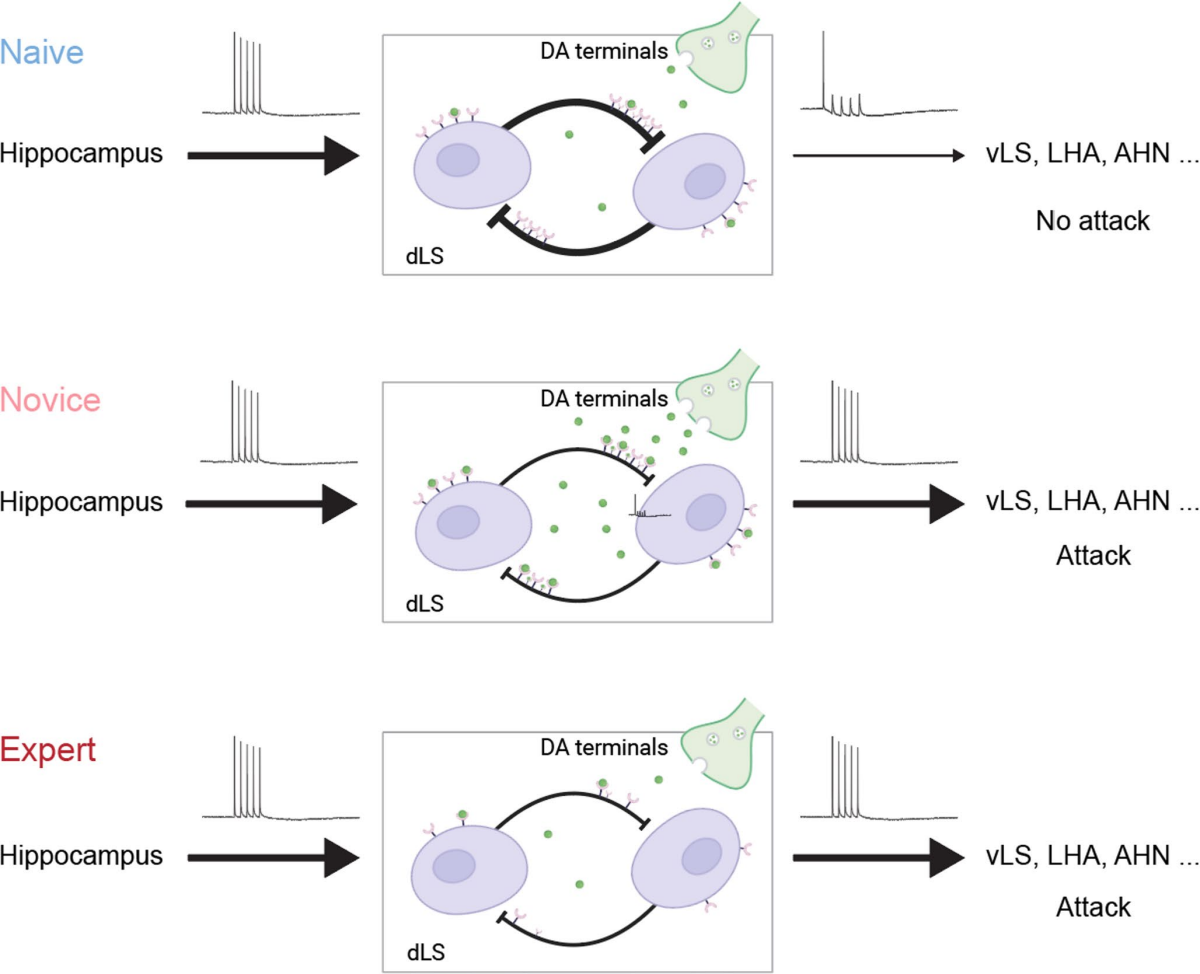
VTA-LS DA facilitates the emergence of male aggression. In naive mice, dLS cells form a strong mutual inhibition network that blocks excitatory hippocampal inputs from reaching downstream regions, thereby preventing the initiation of aggressive behavior. Over male–male interactions, increased DA release in the dLS weakens this local inhibition through the activation of D2 receptors, allowing hippocampal information to pass through and facilitate aggression. As the aggression emerges, the animals are considered novice aggressors. With repeated fighting, the local inhibitory network in the dLS gradually weakens, enabling hippocampal inputs to propagate even without additional DA facilitation. In expert aggressors, DA release during male-male interaction as well as dLS cell responses to DA decrease. dLS: dorsal lateral septum; vLS: ventral lateral septum; LHA: lateral hypothalamus; ANH: anterior hypothalamus. Figure was created using BioRender (https://biorender.com)

Our slice physiology experiments revealed that DA release in the dLS plays a permissive role in aggression by facilitating the flow of aggression-related information through the inhibitory network within the dLS. Under resting conditions, the dLS cells form a dense inhibitory local network that primarily restricts information flow from the hippocampus to downstream areas. DA acts on the densely expressed D2 receptors in the dLS, weakening this mutual inhibition by decreasing presynaptic vesicle release, enabling signals from the hippocampus to pass. In expert aggressors, the mutual inhibition within the dLS is naturally weakened, making the DA assistance less essential (Dai et al. 2025) (Fig. 2).

Many questions remain unanswered regarding the mechanisms underlying DA modulation of aggression. One pressing question is how the experience of aggression downregulates Drd2 mRNA and D2 receptor expression in the dLS. In our study, we observed a significant reduction in Drd2 mRNA expression in the dLS of expert aggressors compared with rookie aggressors (Dai et al. 2025). Interestingly, one study reported that repeated estradiol exposure can decrease Drd2 expression in the striatum (Lammers et al. 1999). However, this effect likely does not directly act on Drd2-expressing striatal cells, as estradiol fails to reduce Drd2 expression in cultured cells. In the case of the LS, the estrogen receptor alpha (Esr1) is predominantly expressed in the ventral LS rather than the dLS (Yao et al. 2023). This suggests a more complex circuit mechanism that may underlie the downregulation of Drd2 expression in the dLS.

Compared with the dense expression of DA receptors, the known DA inputs to the LS appear relatively limited. In our TH staining, we observed sparse tyrosine hydroxylase (TH)-positive terminals in the LS regions, e.g., vLS, that receive no VTA projection (Dai et al. 2025). The origin of these projections and their roles remains unclear. Do they modulate aggression-related circuits directly, or are they involved in other behavioral or physiological processes? Further studies are needed to identify the source of these projections and explore their functional significance in the vLS, particularly in the context of aggression.

### Nucleus accumbens

In the quest to understand DA’s role in aggression, the nucleus accumbens (NAc) has drawn extensive attention due to its dense dopaminergic innervation and its critical role in motivation and reinforcement (Fields et al. 2007; Bromberg-Martin et al. 2010; Beier et al. 2015; Poulin et al. 2018). The NAc can be divided into core and medial shell, each receiving DA inputs from largely non-overlapping VTA DA cells and implicated in different aggression-related functions (Jong et al. 2019).

DA release in NAc core encodes the valence of social experiences. Our previous study demonstrated that positive and negative social experiences evoke opposite DA activity patterns in the NAc core (Dai et al. 2022). Specifically, attacking and winning a social encounter induced a stable increase in DA signals, while being attacked and losing a fight caused a significant decrease in DA levels. We found that optogenetic activation of VTA DA terminals in the NAc core during social defeat alleviated defeat-induced social avoidance (Dai et al. 2022). Willmore et al. selectively stimulated VTA DA terminals in the NAc core during social defeat and reported that the likelihood of fighting back while being attacked significantly increased in the ChR2-expressed mice (Willmore et al. 2022). However, this increase did not depend on the temporal coupling of defeat or fighting with the stimulation, arguing against the role of DA in moment-to-moment action reinforcement, as shown in the dorsal striatum (Willmore et al. 2022; Markowitz et al. 2023). These results collectively suggest that DA release in the NAc core encodes the valence of social experiences, which alters animals’ behavioral reactions to the relevant opponent. Notably, there is little direct evidence supporting the role of NAc core DA in directly promoting attacks.

DA release at the NAc medial shell is seemingly not related to the expression of intermale aggression. Our results, together with a recent report, found that optogenetic activation of VTA DA terminals at Nac medial shell did not promote intermale aggression in novice mice (Mahadevia et al. 2021; Dai et al. 2025). In addition, 6-OHDA lesion of NAc medial shell DA terminals did not affect the emergence of aggression in naïve males (Dai et al. 2025). However, the downstream D1R- and D2R-expressing cells in the NAc medial shell have been implicated in aggression seeking and in the rewarding properties of aggression. Golden et al. trained the aggressive CD-1 mice to lever press for the opportunity to access and attack a weaker opponent (Golden et al. 2019a). In their study, both aggression self-administration and seeking tasks increased Fos expression in D1R- and D2R-expressing cells in the NAc medial shell. Chemogenetic inhibition of D1R cells, but not D2R cells, reduced the frequency of lever pressing (Golden et al. 2019a), suggesting that D1R cell activity in NAc shell is required for aggression seeking. In a related study, Aleyasin et al. found that ΔFosB, a transcription factor known to regulate reward and motivated behaviors, was selectively increased in D1R-expressing cells in the NAc medial shell and core following repeated aggressive encounters (Aleyasin et al. 2018). Genetic manipulation of ΔFosB expression in D1R cells at NAc altered intermale aggression without affecting aggression-induced conditioned place preference (CPP). These findings highlight the complex role of the NAc medial shell in aggression modulation. Rather than acutely controlling the expression of aggressive behavior, DA release in the NAc medial shell may exert long-lasting effects that influence the motivational and reward-related components of aggression through its downstream signaling.

### Medial amygdala

DA signaling in the medial amygdala (MeA), particularly within its posteroventral subdivision (MeApv), plays a role in modulating aggression by balancing social approach and predator avoidance behaviors (Miller et al. 2019). VTA DA cells project to the MeApv, where Drd1 expression is enriched and highly colocalized with excitatory Vglut2 (slc17a6)-expressing populations (Yao et al. 2023). Miller et al. demonstrated that DA inputs to the MeApv regulate approach and avoidance by differentially modulating distinct downstream circuits (Miller et al. 2019). MeApv cells projecting to the bed nucleus of the stria terminalis (BNST) promote social approach, whereas cells projecting to the ventromedial hypothalamus (VMH) facilitate predator avoidance. Activating D1 receptors in the MeApv selectively increases the firing rates of BNST-projecting cells while suppressing the activity of VMH-projecting cells, effectively biasing the system towards social approach and enhancing intermale aggression. Thus, DA input to the MeApv may facilitate social approach preceding attack.

### Prefrontal cortex

The prefrontal cortex (PFC) is crucial for higher-order cognitive functions and is thought to exert top-down control over aggressive behaviors (Lischinsky and Lin 2020; Menon and D’Esposito 2022). A well-known case illustrating this is Phineas Gage, a 19th-century railroad worker who survived a severe injury that damaged his frontal lobes (Damasio et al. 1994). Post-accident, Gage exhibited increased irritability and aggression, illustrating the PFC’s role in restraining such behaviors. In rodent studies, optogenetic activation of excitatory neurons in the PFC has been shown to reduce intermale aggression, further supporting its inhibitory role (Takahashi et al. 2014).

The PFC receives relatively sparse DA inputs from VTA DA cells with high basal firing rate and high bursting properties (Vander Weele et al. 2019). Microdialysis studies have reported that DA concentrations in the PFC rise to approximately 120% following confrontations (Erp and Miczek 2000). Due to its relatively slow dynamics, DA release at PFC may modulate the aggression state — acting as a slow-acting brake that gradually diminishes aggressive actions as the animal establishes dominance —rather than moment-to-moment attack.

Within the PFC, both D1 and D2 receptors are present, but D1 receptor expression is much denser than that of D2 receptors (Yao et al. 2023). Studies have suggested that these receptor populations have distinct functions in social behaviors: for example, D1 receptor-expressing cells are essential for maintaining social rank in tube tests, whereas inhibition of these cells in dominant animals can lower their social status (Xing et al. 2022; Chen et al. 2024). In contrast, direct activation of the Gi signaling pathway in D2 receptor-expressing cells markedly reduces social interaction (Chen et al. 2024). Thus, DA could affect the complex role of PFC in social cognition, such as social interest, learning, recognition, and decision making, although further investigation is needed to clarify the precise role of DA in the PFC.

## Future directions for treating hyperaggression

A key step in treating hyperaggression is to identify effective molecular targets. In our recent study, we found that aggression experience diminishes the role of DA in modulating aggressive behaviors, reducing the efficacy of D2 receptor antagonists in suppressing the behavior (Dai et al. 2025). These observations align with clinical findings suggesting that D2 receptor antagonists may be less effective in hyperaggressive patients with a long history of aggression (Anderson et al. 1984; McCracken et al., 2002; Khushu and Powney 2016; van Schalkwyk et al. 2017, 2018). In this patient population, the aggression-suppressing effect of antipsychotics may be largely due to their impact on the motor system. Atypical antipsychotics, which also antagonize 5-HT2A receptors, appear to suppress aggression with fewer side effects on movement (Ichikawa et al. 2001; Pierre 2005; Sykes et al. 2017). Furthermore, some human studies indicate an association between 5-HT2A receptor expression and high-aggression traits, suggesting a potential causal role in aggression suppression via 5HT2A antagonism (Giegling et al. 2006; Braccagni et al. 2023). However, how serotonin and different 5HT receptors affect aggression requires more investigation.

Efficiently delivering medication to the brain without systemic side effects is another important goal. D2 and 5-HT2A receptors are also expressed outside the brain, such as in immune cells (Herr et al. 2017; Penedo et al. 2021). Consequently, antipsychotics like risperidone and clozapine can suppress immune function, increasing the risk of infections. Orally taken medicines for mental disorders must have the ability to penetrate the blood-brain barrier and will inevitably circulate in the body, affecting many organs beyond the brain. Developing more precise drug delivery methods is crucial for next-generation hyperaggression treatments.

The inherent complexity of the brain poses additional challenges for effective treatment. It is common for the same receptor to be expressed in different brain regions and to be involved in diverse behaviors. For example, D2 receptors are expressed in both the LS and striatum (Yao et al. 2023). Inhibiting D2 receptors in the LS to suppress aggression inevitably also affects D2 receptor-expressing cells in the striatum, leading to compromised movements. In recent years, our understanding of the neural circuits underlying aggressive behaviors has advanced rapidly (Hashikawa et al. 2018; Lischinsky and Lin 2020; Mei et al. 2023). However, identifying non-invasive strategies to selectively target aggression-related regions remains a significant challenge in the field. While enhancer-driven viral strategies have shown promise in achieving high specificity for targeting distinct cell populations (Vormstein-Schneider et al. 2020; Graybuck et al. 2021), implementing permanent interventions like viral infection raises important clinical and ethical concerns. These strategies require careful evaluation to balance potential therapeutic benefits with the long-term implications of irreversible interventions.

Sexual dimorphism of aggression adds another layer of complexity. In many species, males typically show higher levels of aggression than females, a male-biased pattern largely attributed to sexual selection (Lischinsky and Lin 2020). Consequently, rodent studies on aggression, especially those assessing the effectiveness of various anti-aggression medications, have predominantly used males as subjects (Navarro and Manzaneque 1997; Miczek and Tidey 1989; Kudryavtseva et al. 1999; Gendreau et al. 2000). However, recent research indicates that neural circuits generating and modulating aggression are sexually dimorphic (Hashikawa et al. 2018), suggesting that current pharmacological interventions may have different efficacies in treating male and female hyperaggression. Indeed, studies on Drd2 gene polymorphisms have shown that correlations between specific genetic variants and aggression are often stronger in boys than in girls (Guo et al. 2007; Zai et al. 2012). Moreover, our studies in mice found that female aggression elicits lower DA release in the NAc core, and activation of VTA DA cells has no or even a slightly inhibitory effect on female aggression, contrary to their aggression-promoting roles in males (Dai et al. 2022, 2025). These results highlight the pressing need to identify effective therapeutic targets specifically for female aggression.

Genetic, pharmacological, and recent animal circuit studies strongly link DA and aggression. Clinically, the D2 receptor has been a common target for treating hyper-aggression. The neural mechanisms underlying the DA modulation of aggression are starting to emerge and are complex. These understandings will provide critical guidance for designing more patient population-appropriate aggression treatment strategies.

## Supplementary Information

Below is the link to the electronic supplementary material.

Supplementary Material 1 (DOCX 2.87 MB)

## Data Availability

Not applicable.
