# Supplementary material for: Dopamine modulation of aggression
Source: Psychopharmacology (Berl). 2025 Sep 23;243(5):925–48. doi: 10.1007/s00213-025-06893-w (PMC13105275; doi:10.1007/s00213-025-06893-w)
Supplement: Supplementary file 1 — Supplementary Material 1 (DOCX 2.87 MB) [file 213_2025_6893_MOESM1_ESM.docx]

Dear Drs. Jamie Maguire and Marcus Weera,

Thanks for giving us the opportunity to resubmit our review “**Dopamine Modulation of Aggression**” for consideration of publication in *Psychopharmacology*. We greatly appreciate your support.

We have revised the manuscript in accordance with the reviewer’s thoughtful suggestions. We added a new box summarizing the differences between reactive and proactive aggression, along with the behavioral procedures used in mice to study each form. We specified the species used in each cited study and replaced the term “paradigm” with “procedure” throughout. We also corrected and revised sentences related to proactive aggression for clarity.

The revised manuscript now contains 8,692 words, 183 references, 2 figures, and 5 tables.

We hope these changes address the reviewers’ concerns and improve the clarity and completeness of the review. Please let us know if any additional modifications are needed.


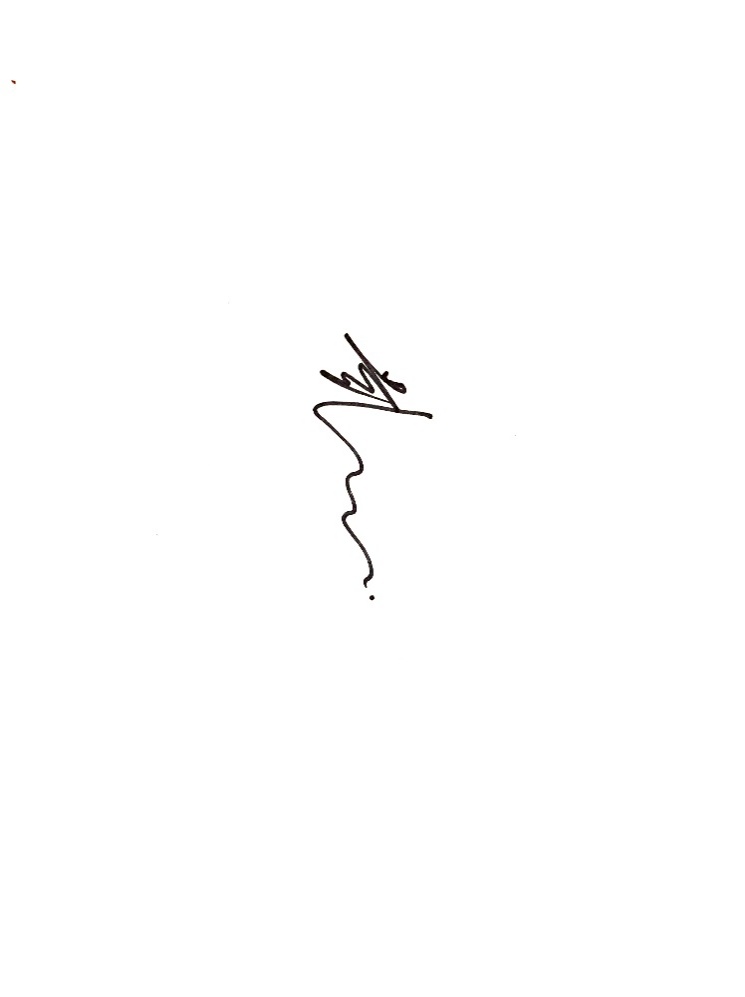


Sincerely yours,

Bing Dai
